# ATLAS2030 Pediatric Gait Exoskeleton: Changes on Range of Motion, Strength and Spasticity in Children With Cerebral Palsy. A Case Series Study

**DOI:** 10.3389/fped.2021.753226

**Published:** 2021-11-24

**Authors:** Elena Delgado, Carlos Cumplido, Jaime Ramos, Elena Garcés, Gonzalo Puyuelo, Alberto Plaza, Mar Hernández, Alba Gutiérrez, Thomas Taverner, Marie André Destarac, Mercedes Martínez, Elena García

**Affiliations:** ^1^Centre for Automation and Robotics, Spanish National Research Council (CSIC-UPM), Madrid, Spain; ^2^Marsi Bionics S.L., Madrid, Spain; ^3^Doctoral Program in Health Sciences, Alcalá de Henares University, Madrid, Spain; ^4^International Doctoral School, Rey Juan Carlos University, Madrid, Spain; ^5^Polytechnic University of Madrid, Madrid, Spain; ^6^La Paz University Hospital, Madrid, Spain

**Keywords:** ATLAS exoskeleton, cerebral palsy, children, range of motion, rehabilitation, robot-assisted gait training, spasticity, strength

## Abstract

**Background:** Cerebral Palsy (CP), the most common motor disability in childhood, affects individual's motor skills, movement and posture. This results in limited activity and a low social participation. The ATLAS2030 exoskeleton is a pediatric device that enables gait rehabilitation for children with neurological or neuromuscular pathologies with gait pathology.

**Purpose:** To study changes in relation to range of motion (ROM), strength and spasticity in children with CP after using the ATLAS2030 gait exoskeleton.

**Methods and Participants:** Three children (mean age 8.0 ± 2.0), two girls and one boy, two of them with GMFCS IV and one with GMFCS III, received robot-assisted gait training (RAGT) with ATLAS2030 for one month.

**Results:** The average time of exoskeleton use was 54.7 ± 10.4 min in all sessions, and all participants were able to perform all exercises. The strength of all muscle groups was increased after the 10 sessions for the participants assessed and the limited ROM in the sagittal plane (hip and knee extension and ankle dorsiflexion) decreased after the use of the exoskeleton compared to the initial state. Spasticity was reduced at the end of the sessions after the use of the exoskeleton compared to their initial state.

**Conclusion:** The ROM, spasticity and strength were improved after RAGT with ATLAS2030 exoskeleton in these children with CP. However, further studies with larger samples should be carried out to confirm our findings.

## Introduction

Cerebral palsy (CP) is the most common cause of chronic childhood motor disability (1), with a prevalence of 2.11 per 1000 live births (2). CP describes a group of permanent disorders affecting movement and posture and causing activity limitations, that are attributed to non-progressive lesions in the developing fetal or infant brain (3). It is a life-long condition that requires regular and continuous care (4). In addition to motor impairment, there are other types of difficulties associated with cognitive or sensory impairment in this population (3).

The presentation of CP and the individual context vary greatly among individuals with CP, therefore, effective management of the condition requires personalized and well-coordinated care (3).

Improving walking capability is one of the intervention objectives in children with CP. To achieve or improve the gait pattern, infants require appropriate muscle strength, nerve innervation, good endurance, and less fatigue (5). Walking is more effortful for children with CP than it is for their non-disabled peers due to their motor impairments, such us altered range of motion, weakness and spasticity (6). Walking devices can improve endurance, stability, and posture (5).

Robot-assisted gait training (RAGT) is a new global physiotherapy technology that applies robotics knowledge, along with high-intensity repetitive exercises, to improve mobility of patients with neurological disorders (7). The use of robotic trainers for gait rehabilitation in several motor diseases has increased in the last decades, both in adults and children (8). In the future, it is expected that the exoskeletons will be further developed to be integrated as a means of mobility in the daily life of children with gait disorders. This requires that future exoskeletons must not be fixed to an installation, but wearable.

In recent years, the literature on pediatric walking exoskeletons has increased significantly (9). Several models of pediatric exoskeletons are found in the scientific literature used in children with CP, of which, only two are wearable or portable. The HAL® (10), which in the case of being used in children, it would need the addition of an external adaptation (11) to the device that supports the participant's weight. The other one is the CPWalker (12), which is still in the experimental phase and not yet available for commercialization.

The Lokomat® is another exoskeleton worth mentioning due to its widespread use and large number of publications in the scientific literature (13). It is not considered a portable exoskeleton because, transporting them from one place to another is quite challenging due to their weights and sufficient space is required. In addition, the use of a treadmill is required for the use of the Lokomat®. The ATLAS2030 exoskeleton (Marsi Bionics, Madrid, Spain), (14) which has recently obtained the European certificate for its commercialization, seems to offer promising results in the field of walking exoskeletons aimed at the pediatric population, in comparison with the exoskeletons presented above.

**Figure 1:**
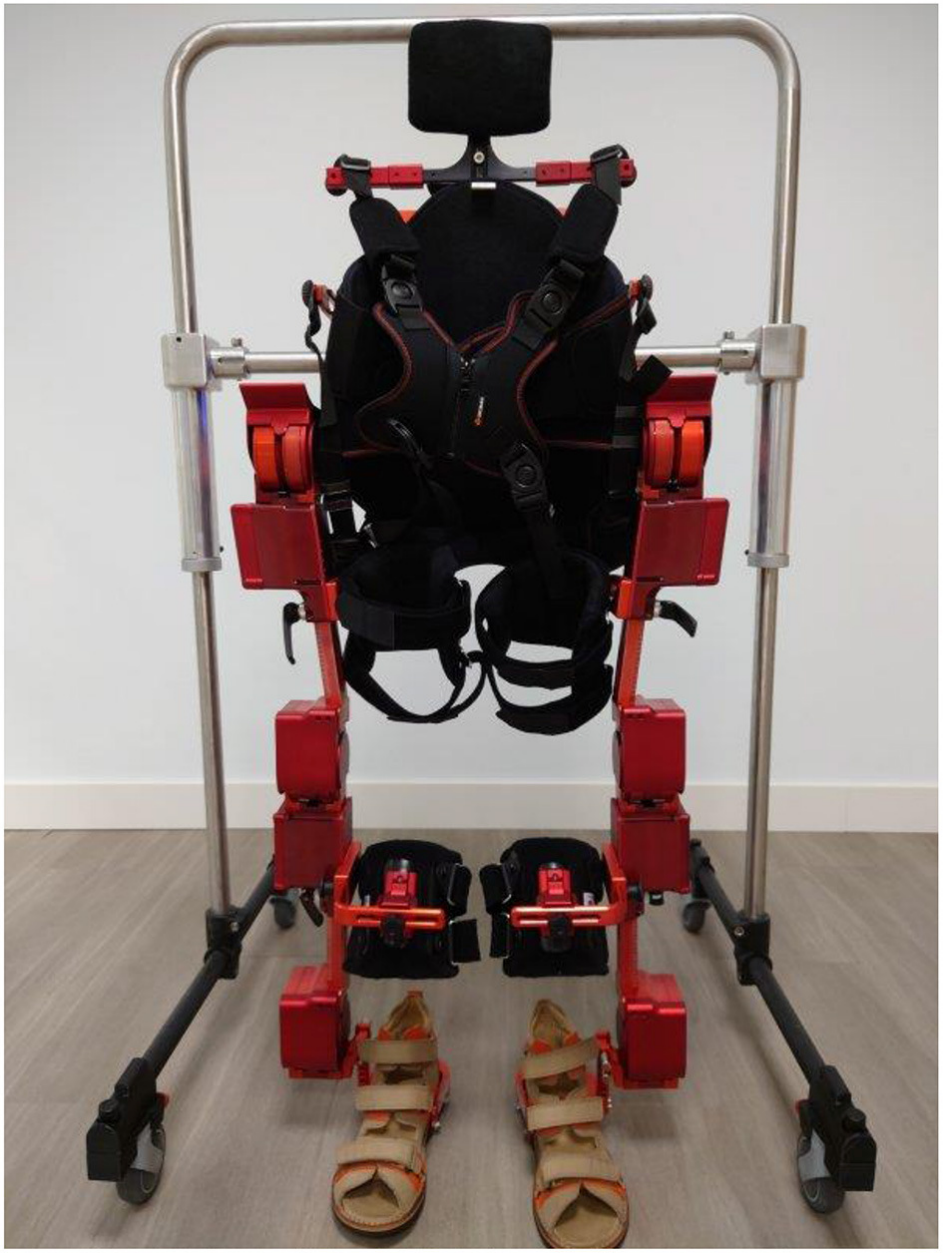
ATLAS2030 exoskeleton.

ATLAS2030 exoskeleton (Figure 1) is a bilaterally driven wearable exoskeleton which offers eight actuated degrees of freedom (DoFs), four per leg (hip, knee and ankle rotations in the sagittal plane, and hip rotation in the frontal plane). These actuators are based on the patented technology ARES (15), offering adjustable stiffness with the ability to minimize forces due to impacts, safely interacting with the user, and storing and releasing energy in passive elastic elements. ATLAS2030 exoskeleton can be adapted to the anthropometric characteristics of the individual children by adjusting the geometry of the exoskeleton and the position of the leg cuffs, and setting specific angular limitations of the lower limb joints. To enhance the safety of the patient and to decrease the physical involvement of the physiotherapists, it has a safety frame with swivel wheels attached to the exoskeleton.

ATLAS2030 exoskeleton allows walking forward and backward and it offers two modes of use: (1) automatic mode in which it assists patient's gait completely following a reference pattern based on the kinematics of healthy subjects at the set speed and (2) active mode in which the movement stops during the swing phase until the patient exerts a certain threshold joint force to continue the movement. It also allows movements from sitting to standing (sit-to-stand). All these features are controlled from an application running on a tablet linked to the Wi-Fi connection provided by the exoskeleton.

The ATLAS active lower-limb exoskeleton, designed specifically for Spinal Muscular Atrophy (SMA) type II kids, provides intensive walking exercise in order to improve the patient's health, enhance the overall motor system, delay and reduce the onset of scoliosis and increase the quality of life and life expectancy (14). In a preliminar published study (14), using a model prior to the ATLAS2030 (the ATLAS2020), its safety, as well as other clinical variables, were proven. Upper limb strength and respiratory muscle strength were assessed, and showed functional improvements in SMA patients. Both parameters were selected because they are usually found to be altered due to muscle degeneration caused by the SMA pathology.

Prior to this research, a pilot study was conducted to measure the feasibility and adaptability of using the ATLAS2030 in the pediatric population with CP, as well as a safety and usability study. Data of both are not currently accessible in the literature, but showed positive results.

In CP, the damage in the child's Central Nervous System (CNS) shows a different symptomatology than SMA. Alterations in the range of motion, strength and muscle tone are frequent in these children, and can lead to difficulties or the impossibility of walking (12). Since the ATLAS2030 exoskeleton is a recently created device, no previous studies have been performed in which these variables have been studied.

Based on the effects that the use of the ATLAS2030 exoskeleton has had on children with gait impairment, and the symptomatology of children with CP, the following hypothesis is presented: “*The use of the ATLAS2030 exoskeleton produces improvement in lower limb range of motion limitation, strength and spasticity in a sample of children with CP*”.

In order to evaluate the occurrence of changes before and after using the ATLAS2030 exoskeleton in a population of children with CP, the main objectives of this article are:

(1) To assess changes in range of motion in the lower extremities, in those movements that are affected in children with CP.
(2) To measure increase or decrease in the strength of the muscles responsible for lower extremity movement.
(3) To evaluate the spasticity of the different movements in the musculature of both legs.

## Materials and Methods

### Participants

Participants were selected from the database of La Paz University Hospital (Madrid, Spain) by an experienced physician, on the following inclusion criteria: Children aged from 3 to 14 years old with diagnosis of CP in GMFCS levels III-V (inability to walk without assistive device), whose parents or legal guardians had signed the informed consent. The exclusion criteria were those related to the use of the device (Table 1).

**Table 1:** Exclusion criteria for use of the device.

**Exclusion criteria for the use of ATLAS2030 exoskeleton**
• Weight over 40 kg. • Femoral length (from hip joint to knee joint in the sagittal plane) smaller than 22 cm or larger than 38 cm. • Tibial length (from knee joint to ankle joint in the sagittal plane) smaller than 21 cm or larger than 37 cm. • Distance between trochanters smaller than 24 cm or larger than 40 cm. • The inability to understand simple instructions, to report basic needs or to actively collaborate in the therapy. • Needing invasive or non-invasive daytime ventilation. • Suffer from orthostatic hypotension. • Limitation of hip or knee passive extension range of movement higher than 20 degrees. • Cobb angle higher than 25 degrees without the possibility of wearing a brace during the test. • Severe skin alteration on the lower extremities. • Surgical intervention scheduled (spine, extremities) within the next 6 months, or surgery performed (spine, extremities) within the last 6 months. • History of fracture without trauma. • History of traumatic bone fracture in the lower limbs or pelvic girdle within the last 3 months. • No regular standing rehabilitation sessions. • Lack of head or trunk control in an upright position without the possibility of wearing a brace during the use of the exoskeleton. • Refusal of the patient or legal guardian to include the child in the study. • Skin problems (diseases, allergies, sensitivity, etc.) that prevent the use of the exoskeleton accessories on the patient's skin.

This study was performed in accordance with the Declaration of Helsinki (16), approval was obtained (reference 47/370329.9/19) by Comunidad de Madrid Regional Research Ethics Committee with Medical Products and all parents, or legal guardians, of the participants gave written informed consent. The clinical trial has been registered on Clinical Trials.gov: NCT04837157.

### Outcome Measures

The outcome measures assessed were: limitation of range of motion, strength and spasticity of both lower limbs. For the assessment of range of motion (ROM) limitation, a specialized physiotherapist used a manual goniometer and followed the rules for its correct use in an accurate manner. To assess the improvements of the use of the exoskeleton, the strength of the lower limbs was measured using a Hand-held Dynamometer (HHD) (17). The Modified Ashworth Scale (MAS) (18) was used to evaluate spasticity. To avoid inter-rater bias, all measurements were performed by the same evaluator on all participants and measurements. The movements analyzed, for all variables, were hip flexion-extension and abduction, knee flexion-extension and ankle dorsi and plantarflexion. Hip adduction was not measured as subluxation are commonly found in children within this population. Spasticity was measured at each session before and after device use to assess whether any change was observed after the use of ATLAS2030 exoskeleton. ROM were measured at baseline, endpoint and there were two mid-study assessments. Force measurements were performed in conjunction with ROM measurements. Before recording the force values obtained, a test measurement was performed with all the children to ensure that they had understood the instructions correctly and performed the correct movement.

### Design of the Study

The study was performed in Madrid at Marsi Care research facilities, located at the Center for Automation and Robotics (CAR) from the Spanish National Research Council and Technical University of Madrid (CSIC-UPM). The study consisted of 10 sessions, organized as follows: one telephone screening visit (V0), one laboratory inclusion visit (V1), eight bi-weekly rehabilitation visits with the device (V2-V9), in which, in two of them (V5 and V9) the participants were also remeasured, and a final reassessment visit (V10).

During the telephone screening visit (V0), the first contact was made with some families from La Paz University Hospital database, and it was confirmed that they met the main inclusion criteria such as diagnosis of the pathologies, age and GMFCS level. Once confirmed that they met the inclusion criteria, both parents or legal guardians and the Principal Investigator signed the informed consent form.

During the inclusion visit (V1), it was assessed that the participants did not meet any of the exclusion criteria that would prevent them from using the device, as well as the appropriate anthropometric measurements to be able to adjust the geometry of the ATLAS2030 to the characteristics of the children. The first measurements of ROM, strength and spasticity were taken, and a test of adjustment and adaptation to the device was carried out so that the children could adapt to its use.

Rehabilitation sessions (V2-V9) were biweekly every other day. The participants used the exoskeleton a maximum of 60 minutes to perform six activities. These activities comprised: (1) non-walking standing position, (2) sit-to-stand exercise using that mode of the exoskeleton, (3) walking forward and backward using both the automatic and active modes of use of the exoskeleton, (4) trunk rotations while walking in automatic mode, (5) ball or balloon games while walking in automatic mode and (6) balance exercises holding the standing position. The walking area was 10 m x 6 m, providing the possibility to change directions to not only walk in a straight line, thanks to the swivel wheels of the device's safety frame. Rest periods were allowed if required by the participants. Sessions V5 and V9 were considered control sessions, and in addition to the use of the device, ROM and strength measurements were taken.

The last visit (V10) was performed 48 h after the last session with the exoskeleton, and to assess short-term changes that the use of the device may have produced on the limitations of ROM, strength and spasticity.

In all sessions using the device, the patient's measurements were placed on the exoskeleton, lower limb muscle stretching was performed for the children's comfort prior every session, and spasticity was measured before and after using the device.

### Statistical Analysis

As the sample size was three participants, it was considered to carry out a descriptive analysis of all quantitative data. And secondly, in order to objectively measure the patient's evolution, it was decided to evaluate the progression of the therapy by comparing each patient to him-/herself by means of averages and standard deviations. All analyses and graphics were performed using IBM^®^ SPSS^®^ Statistics v27 software (IBM Corporation, Armonk, NY, USA).

## Results

Three patients (2 girls, 1 boy) with CP were recruited to participate in this study. They were 8.0 ± 2.0 years old, 124.67 ± 17.91 cm tall and weighed 20.0 ± 5.0 kg. Details about the patients' characteristics are listed in Table 2. All three participants were habitual wheelchair users.

**Table 2:** Patients' description.

**Patient**	**Disease**	**GMFCS(level)**	**Walking support**	**Age (years)**	**Height (cm)**	**Weight (kg)**
1	Spastic and dystonic tetraparesis - CP	IV	WC	8	135	20
2	Spastic tetraparesis - CP	IV	WC	6	104	15
3	Spastic tetraparesis - CP	III	WC and walker	10	135	25

*CP, Cerebral Palsy; GMFCS, Gross Motor Function Classification System; WC, wheelchair*.

### Patient 1

A 10-year-old boy diagnosed with spastic tetraparesis of unknown origin. GMFCS IV, self-propelled wheelchair for mobility user. Mild-moderate spasticity in lower limbs [overall pattern of 1+ according to the Modified Ashworth Scale (MAS)]. He has done exoskeleton gait therapy with ATLAS2030 before, during a single session (NCT04813601).

### Patient 2

An 8-year-old girl diagnosed with spastic-dystonic tetraparesis due to perinatal hypoxia. GMFCS IV, manual wheelchair user. She has moderate spasticity (Global pattern of 2 according to the MAS), and she has never received botulinum toxin. She has never received walking therapy with an exoskeleton.

### Patient 3

A 6-year-old girl diagnosed with spastic tetraparesis due to periventricular leukomalacia. She has GMFCS III and uses a manual wheelchair and walker. She presents moderate spasticity (Global pattern of 1 according to the MAS), and used to receive botulinum toxin every 6 months. She has never received walking therapy with an exoskeleton.

All participants successfully completed the 10 sessions, and they used all exoskeleton's functions. The average time of use per session was 54.7 ± 10.4 minutes. Of those, (1) 10.0 ± 0.3 minutes non-walking standing position; (2) 10 ± 0.0 minutes were spent performing sit-to-stand exercise; (3) 19.9 ± 2.5 minutes walking; (4) 9.9 ± 0.6 doing trunk rotations while walking; (5) 4.7 ± 1.9 minutes doing balloon games while walking in automatic mode and (6) 4.7 ± 1.9 minutes performing balance exercises holding static position. All participants were able to perform all exercises and use all modes of the exoskeleton, regardless of their GMFCS level.

The average spasticity was measured for both legs of the three patients for the different movements assessed at the beginning and at the end of every session. Overall, spasticity was reduced at the end of the sessions after the use of the exoskeleton compared to their initial state (before 0.6 ± 0.6, after 0.5 ± 0.5). The average scores of each participant can be seen in Table 3. In addition, Table 4 shows the data of spasticity assessments at the first visit and at the last visit of the study by joint and movement.

**Table 3:** Average spasticity measured for both legs of the three patients for the different movements assessed at the initial (V1) and final (V10) visits of the study, measured by the MAS.

	**Hip**						**Knee**				**Ankle**					
	**Flexion**		**Extension**		**Abduction**		**Flexion**		**Extension**		**Dorfx**		**Plantarfx**		**TOTAL**	
**Visit**	**V1**	**V10**	**V1**	**V10**	**V1**	**V10**	**V1**	**V10**	**V1**	**V10**	**V1**	**V10**	**V1**	**V10**	**V1**	**V10**
Average	0.9	0.1	1.0	0.1	0.7	0.2	0.7	0.1	1.5	0.4	1.3	0.2	1.0	0.0	1.0	0.1
SD	0.9	0.4	0.8	0.3	0.6	0.5	0.7	0.3	0.9	0.6	1.0	0.5	0.9	0.0	0.8	0.4

*Dorfx, dorsiflexion; plantarfx, plantarflexion; V, visit; SD, standard deviation*.

**Table 4:** Average spasticity measured for both legs of the three patients for the different movements assessed at the beginning (Before) and at the end (After) of every session after the use of the exoskeleton, measured by the MAS.

	**P1**		**P2**		**P3**	
	**Before**	**After**	**Before**	**After**	**Before**	**After**
Hip flexion	1.1 ± 0.7	0.9 ± 0.6	0.5 ± 0.4	0.4 ± 0.4	0.7 ± 0.4	0.7 ± 0.6
Hip extension	0.9 ± 0.7	0.7 ± 0.4	0.3 ± 0.7	0.4 ± 0.5	0.4 ± 0.4	0.2 ± 0.4
Hip abduction	1.0 ± 0.8	0.8 ± 0.6	0.4 ± 0.4	0.3 ± 0.3	0.9 ± 0.4	0.7 ± 0.4
Knee flexion	0.9 ± 0.6	0.2 ± 0.8	0.5 ± 0.5	0.3 ± 0.4	0.6 ± 0.4	0.5 ± 0.4
Knee extension	1.4 ± 0.7	0.6 ± 0.5	0.7 ± 0.9	0.6 ± 0.6	0.6 ± 0.3	0.6 ± 0.4
Ankle dorfx	0.3 ± 0.4	0.3 ± 0.4	0.8 ± 0.8	0.5 ± 0.3	0.9 ± 0.8	0.8 ± 0.8
Ankle plantarfx	0.4 ± 0.4	0.4 ± 0.4	0.4 ± 0.7	0.1 ± 0.3	0.3 ± 0.4	0.1 ± 0.2

*Dorfx, dorsiflexion; plantarfx, plantarflexion; P, patient*.

**Figure 2:**
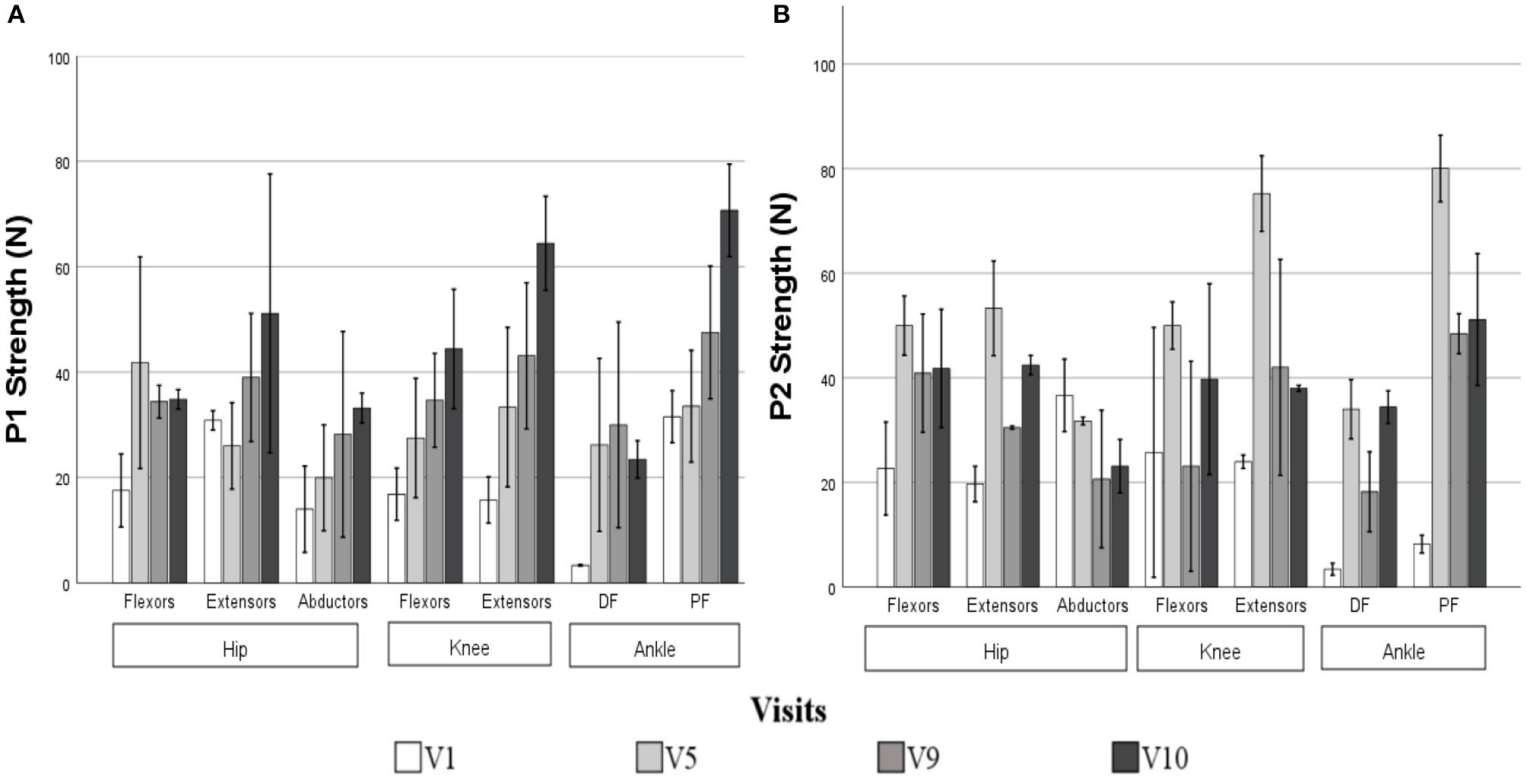
Strength measurements collected for patient 1 **(A)** and 2 **(B)** for the different movements assessed in the control visits measured with a Hand-Held Dynamometer in Newtons. Error bars at 95 % CI show a standard deviation of the data. DF, Dorsiflexion; PF, Plantarflexion.

The strength was measured for two participants, since one of them struggled in understanding the measurement instructions. The greatest increases in muscle strength were notable between sessions V1-V5 for hip flexors, knee extensors and ankle dorsiflexors and plantarflexors. On the other hand, in hip extensors and knee flexors the differences were most notable between V5-V9. The strength of all muscle groups increased after the 10 sessions for both participants assessed. All information regarding the strength data is reflected in Figure 2, Table 5. In the first strength assessment there were larger deviations from the final assessments as can be seen in Table 5.

**Table 5:** Average strength muscle in different visits. Measured with Hand Held Dynamometer (N).

**Strength (N)**	**V1 (First evaluation)**	**V10 (Last evaluation)**
Hip flexion	20.1 ± 3.6	42.4 ± 10.7
Hip extension	25.3 ± 7.9	52.2 ± 1.5
Hip abduction	25.3 ± 16.0	32.5 ± 1.0
Knee flexion	21.3 ± 6.3	47.2 ± 3.9
Knee extension	19.8 ± 5.8	69.8 ± 7.6
Ankle dorsiflexion	3.4 ± 0.0	28.7 ± 7.5
Ankle plantarflexion	19.9 ± 16.5	75.4 ± 6.6

**Figure 3:**
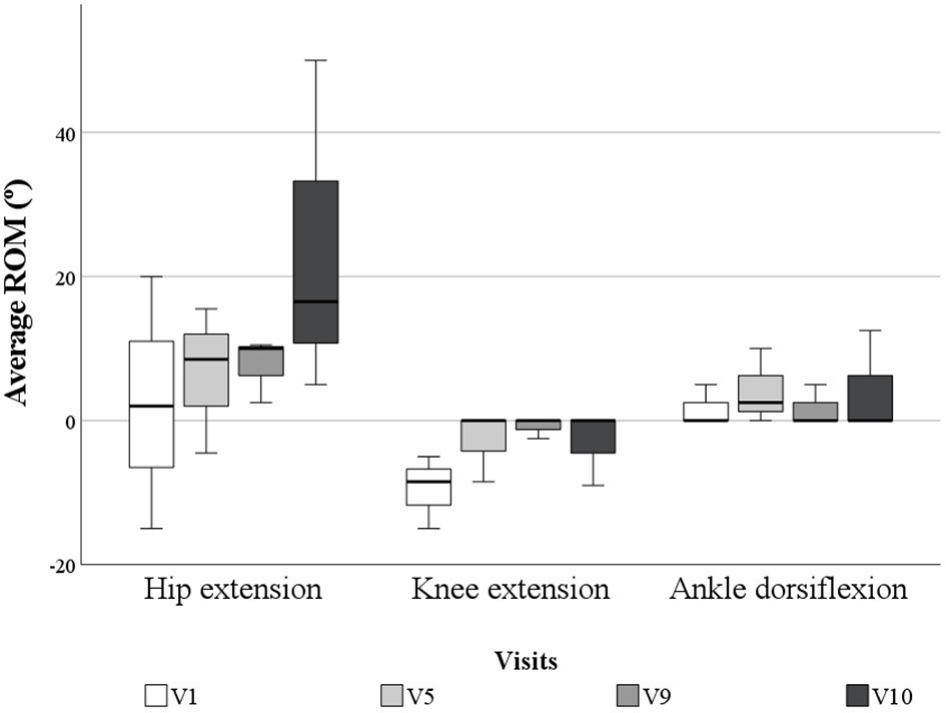
Average ROM of the hip and knee extension and ankle dorsiflexion in degrees for all the patients during the control visits.

Regarding the ROM, all of them (hip extension, knee extension and ankle dorsiflexion) improved after the use of the exoskeleton (Figure 3). The mean hip extension at the start of the study was 2.3 ± 17.5 degrees, while at the end of the study it increased to 23.8 ± 23.4 degrees. On the other hand, at the beginning of the study the average knee extension was −9.5 ± 5.1 degrees, while at the last session it was −3.0 ± 5.2. Finally, the average ROM of dorsal ankle flexion was 1.7 ± 2.9 degrees, while at the end of the study it was 4.2 ± 7.2 degrees.

## Discussion

CP is the most common cause of motor disability in childhood. Due to the predominant motor symptomatology in these children, the use of assistive technologies and other mobility aids are very useful both for therapists and for the positive effects on the children. In Novak's review (19), treatments for children with CP are classified according to whether or not they are recommended in the literature. Walking aids are highly recommended to improve walking speed, walking endurance and gross motor in children with CP (19).

The use of Assisted Technology (AT) is recommended for children with CP, both individually and in conjunction with adaptive equipment or virtual reality, to improve hand function, mobility and gait (19). The ATLAS2030 pediatric exoskeleton is a robotic device for children with CP, which can be used both for gait rehabilitation and as a tool for performing physical activity. In addition, strength can be worked out by requiring the children to make an active isometric contraction to initiate the gait while using “the active mode”.

In the last decade, the paradigm around disability has been shifting, with the focus on improving functionality and participation rather than on symptomatology or diagnoses of the disease. The International Classification of Functioning, Disability and Health (ICF) (20) was created to provide a unified and standardized language and conceptual framework for the description of health and health-related conditions. Devices such as the ATLAS2030 exoskeleton could facilitate mobility for patients with a restricted one, based on the ICF biopsychosocial model of disability. CP is a pathology that, in almost all cases, usually affects function (neuromusculoskeletal and movement-associated functions, and other body functions in children with comorbidity or greater involvement), activities and participation, and environmental factors (if they use a wheelchair to move around or other support products). Through the use of the evaluated device, and in accordance with the ICF, interventions for the improvement of function, activities and participation such as lower limb strengthening, sit-to-stand training, standing posture maintenance, and gait training could be implemented to improve the functionality of children with CP.

This study was the first to measure changes in joint ROM, strength and spasticity in children with CP after using the ATLAS2030 exoskeleton over time. Other publications in the literature have measured changes in these variables after RAGT therapy with different devices. Amman-Reiffer et al. (21) found no significant difference in spasticity measured by MAS between the use of conventional physiotherapy and RAGT therapy on the same measure, compared to twice as much conventional physiotherapy as RAGT, using the Lokomat® exoskeleton for 45 min, in a sample of 16 participants. In the study by Digiacomo et al. (22), no significant differences in MAS were observed after using the Lokomat® Nanos exoskeleton for 4 weeks in 60-min sessions in a sample of 14 participants. In the study by Peri et al. (23) there were no differences in spasticity measured with the Ashworth scale between single treatment with conventional physiotherapy, single treatment with RAGT therapy for 4 weeks, using the Lokomat® exoskeleton for 30 min, or combining both therapies, in a sample of 44 children with CP. On the contrary, in this work, there is an improvement can be seen in spasticity, before and after using the device, in 2 of the 3 children, those with a higher level of severity in GMFCS. Spasticity decreased in all movements between before and after use of the exoskeleton, showing a decrease in hip extension. Regarding the movements, all joint movements evaluated showed a decrease in the MAS scale.

Bayón et al. (12) found an improvement in maximal isometric strength using the CPWalker exoskeleton over 8 weeks for 60 min in a sample of 4 participants. Kuroda et al. (10) found a decrease in maximal isometric strength in knee extension using the Hybrid Active Limb (HAL®) exoskeleton in a single participant in 20-min sessions over 4 weeks. Regarding the measurement of strength in the present study, a progressive increase was observed throughout the four control sessions, with a notable increase between the final session and the 48-h follow-up, where there was a notable difference even though the device was not used between these sessions. Despite the fact that the measurement of strength using the HHD is validated for use in children with CP (17), a “learning bias” may occur during the performance of this test, as well as the children's own motivation as it is the last session. This could explain the presence of larger deviations in the initial measurement (V1) versus the final strength measurement (V10). This fact would favor participants performing the test better each time they perform it, explaining the increase in strength between the final session and the control session, despite not having received therapy between these. To avoid these biases, it would have been interesting to use the Gold Standard measure for strength: the isokinetic dynamometer (24).

Amman-Reiffer et al. (25) found no significant difference in ROM between using conventional physiotherapy and RAGT therapy in children with CP. In this work, an improvement in ROM was observed between the initial session and the end of the study.

Studies with walking exoskeletons to date are mostly Non-randomized Intervention Studies (NRSIs), since these types of interventions are not yet widely used in rehabilitation centers due to their novelty. For this reason, this study was a prospective pilot study with a number of potential limitations, including a small number of participants from a single geographic location and a lack of control group, inherent in a pilot study. Because of that, researchers should be cautious to generalize the results of this study and, thus, future studies should be conducted to assess the safety and effectiveness of the device. Based on the data obtained in one month of treatment, and in the absence of a change in trend in the progression of the variables, it is estimated that there would continue to be an improvement in the variables studied over a longer period of time. In addition, an adequate sample size would provide more evidence for the results obtained.

Due to the limited size of the sample presented here, and the wide clinical variability of CP, it has not been possible to collect the benefits of using the ATLAS2030 exoskeleton in patients with all symptomatology. Therefore, it would be interesting for future studies to include participants from all levels of GMFCS classification, as well as greater typology in the diagnosis of CP (ataxia or athetosis). In addition, by including participants with low GMFCS (levels I-III) who are able to walk, the effects of using the ATLAS2030 exoskeleton on gait parameters could be studied. In the same way, it would be interesting to introduce other functional tests to evaluate changes in the use of the ATLAS2030 exoskeleton in children with CP on these dimensions. The ATLAS2030 could be included as part of the rehabilitation programs for children with CP as it is certified for commercialization.

This is the first study to analyze changes following the use of the ATLAS2030 exoskeleton, including physical variables, which are the most frequently limited in children with cerebral palsy. The main limitation of the study is the small sample size, so it would be ideal to carry out a similar study but with a larger sample size, which would allow extrapolation of the results to the population studied. Additionally, the retention time of the treatment was not evaluated.

## Conclusions

This case series study shows improvements, after receive RAGT therapy with ATLAS2030 exoskeleton in children with CP. Spasticity was significantly reduced with changes found between the beginning and the end of the study, and before and after use of the exoskeleton in each session. In addition, strength and ROM were increased in all children. Because of that, this research could serve as preliminary support for future clinical integration of ATLAS2030 as a part of rehabilitation in children with CP.

## Data Availability Statement

The raw data supporting the conclusions of this article will be made available by the authors, without undue reservation.

## Ethics Statement

The studies involving human participants were reviewed and approved by Comunidad de Madrid Regional Research Ethics Committee with Medical Products (Reference 47/370329.9/19). Written informed consent to participate in this study was provided by the participants' legal guardian/next of kin. Written informed consent was obtained from the minor(s)' legal guardian/next of kin for the publication of any potentially identifiable images or data included in this article.

## Author Contributions

ED, CC, and JR wrote the whole manuscript and contributed to the study's clinical tasks and data collection. EGarcé, GP, and TT contributed to conception and design of the study. MM and EGarcí were the principal investigators of the study. AP, MH, AG, and MD contributed to the revision of the article.

## Funding

This work has been partially funded by Agencia Estatal de Investigación through Grant PID2019-110492RB-I00 and from Comunidad de Madrid through Grant IND2018/TIC9618. AP Acknowledges support from Comunidad de Madrid through Grant IND2017_TIC-7698. JR acknowledges support from Consejería de Educación e Investigación de la Comunidad de Madrid through Grant PEJ-2018-AI_TIC-11333. CC acknowledges support from Consejería de Educación e Investigación de la Comunidad de Madrid through Grant PEJ-2019-AI_TIC-15202. GP acknowledges support from Agencia Estatal de Investigación (Ministry of Science and Innovation) through Grant DI-16-08731. EGarcé acknowledges support from Comunidad de Madrid through Grant IND2018/TIC9618. MD acknowledges support from Agencia Estatal de Investigación (Ministry of Science and Innovation) through Grant PTQ2018-010119.

## Conflict of Interest

EGarcé, GP, AP, TT, and MD belong to the research or clinical team of the company developing the device. The remaining authors declare that the research was conducted in the absence of any commercial or financial relationships that could be construed as a potential conflict of interest.

## Publisher's Note

All claims expressed in this article are solely those of the authors and do not necessarily represent those of their affiliated organizations, or those of the publisher, the editors and the reviewers. Any product that may be evaluated in this article, or claim that may be made by its manufacturer, is not guaranteed or endorsed by the publisher.

## References

[1] Pakula AT Van Naarden Braun K Yeargin-Allsopp M. Cerebral palsy: classification and epidemiology. Phys Med Rehabil Clin N Am. (2009) 20:425–52. 10.1016/j.pmr.2009.06.001 19643346

[2] Sellier E Platt MJ Andersen GL Krägeloh-Mann I De La Cruz J Cans C. Decreasing prevalence in cerebral palsy: a multi-site European population-based study, 1980 to 2003. Dev Med Child Neurol. (2016) 58:85–92. 10.1111/dmcn.12865 26330098

[3] Bax M Goldstein M Rosenbaum P Leviton A Paneth N Dan B. Proposed definition and classification of cerebral palsy, April 2005. Dev Med Child Neurol. (2005) 47:571. 10.1017/S001216220500112X 16108461

[4] Nzovak I. Evidence-based diagnosis, health care, and rehabilitation for children with cerebral palsy. J Child Neurol. (2014) 29:1141–56. 10.1177/0883073814535503 24958005

[5] Rodríguez-Costa I De La Cruz-López I Fernández-Zárate I Maldonado-Bascón S Lafuente-Arroyo S Nunez-Nagy S. Benefits of a low-cost walking device in children with cerebral palsy: a qualitative study. Int J Environ Res Public Heal Artic. (2021) 18:2808. 10.3390/ijerph18062808 33801985 PMC7998765

[6] Johnston TE. Energy cost of walking in children with cerebral palsy: relation to the gross motor function classification system. Dev Med Child Neurol. (2004) 46:575. 10.1017/S0012162204000064 15287251

[7] Chung BPH. Effectiveness of robotic-assisted gait training in stroke rehabilitation: a retrospective matched control study. Hong Kong Physiother J. (2017) 36:10–6. 10.1016/j.hkpj.2016.09.001 30931034 PMC6385094

[8] Cumplido C Delgado E Ramos J Puyuelo G Garcés E Destarac MA. Gait assisted exoskeletons for children with cerebral palsy or spinal muscular atrophy: a systematic review. Neurorehabilitation. (2021). 10.3233/NRE-210135 34219676

[9] Lefmann S Russo R Hillier S. The effectiveness of robotic-assisted gait training for paediatric gait disorders: systematic review. J Neuroeng Rehabil. (2017) 14:1–10. 10.1186/s12984-016-0214-x 28057016 PMC5217646

[10] Kuroda M Nakagawa S Mutsuzaki H Mataki Y Yoshikawa K Takahashi K. Robot-assisted gait training using a very small-sized Hybrid Assistive Limb® for pediatric cerebral palsy: a case report. Brain Dev. (2020) 42:468–72. 10.1016/j.braindev.2019.12.009 32249081

[11] Ceiling Hoist Solutions & Patient Handling Equipment | HLS Healthcare.

[12] Bayón C Martín-Lorenzo T Moral-Saiz B Ramírez Ó Pérez-Somarriba Á Lerma-Lara S. A robot-based gait training therapy for pediatric population with cerebral palsy: Goal setting, proposal and preliminary clinical implementation. J Neuroeng Rehabil. (2018) 15:1–15. 10.1186/s12984-018-0412-9 30053857 PMC6063005

[13] Nam KY Kim HJ Kwon BS Park JW Lee HJ Yoo A. Robot-assisted gait training (Lokomat) improves walking function and activity in people with spinal cord injury: a systematic review. J Neuroeng Rehabil. (2017) 14:1–13. 10.1186/s12984-017-0232-3 28330471 PMC5363005

[14] Sanz-Merodio D Puyuelo G Ganguly A Garces E Goñi A Garcia E. EXOtrainer project clinical evaluation of gait training with exoskeleton in children with spinal muscular atrophy. In: Springer Tracts in Advanced Robotics. Springer (2020) vol. 132, p. 211–27. 10.1007/978-3-030-22327-4_10

[15] Cestari M Sanz-Merodio D Garcia E. A new and versatile adjustable rigidity actuator with add-on locking mechanism (ARES-XL). In: Actuators (2018). Vol. 7, p. 1. 10.3390/act7010001

[16] Declaración de Helsinki de la AMM – Principios éticos para las investigaciones médicas en seres humanos – WMA – The World Medical Association. Available online at: https://www.wma.net/es/policies-post/declaracion-de-helsinki-de-la-amm-principios-eticos-para-las-investigaciones-medicas-en-seres-humanos/. (accessed September 16, 2021)

[17] Crompton J Galea MP Phillips B. Hand-held dynamometry for muscle strength measurement in children with cerebral palsy. Dev Med Child Neurol. (2007) 49:106–11. 10.1111/j.1469-8749.2007.00106.x 17253996

[18] Mutlu A Livanelioglu A Gunel MK. Reliability of Ashworth and Modified Ashworth Scales in children with spastic cerebral palsy. BMC Musculoskelet Disord. (2008) 9:44. 10.1186/1471-2474-9-44 18402701 PMC2330046

[19] Novak I Morgan C Fahey M Finch-Edmondson M Galea C Hines A. State of the evidence traffic lights 2019: systematic review of interventions for preventing and treating children with cerebral palsy. Pediatr Neurol. (2020) 20:3. 10.1007/s11910-020-1022-z 32086598 PMC7035308

[20] International Classification of Functioning Disability and Health (ICF). https://www.who.int/standards/classifications/international-classification-of-functioning-disability-and-health. (accessed July 12, 2021)

[21] Ammann-Reiffer C Bastiaenen CHG Meyer-Heim AD van Hedel HJA. Effectiveness of robot-assisted gait training in children with cerebral palsy: a bicenter, pragmatic, randomized, cross-over trial (PeLoGAIT). BMC Pediatr. (2017) 17:1–9. 10.1186/s12887-017-0815-y 28253887 PMC5333417

[22] Digiacomo F Tamburin S Tebaldi S. Improvement of motor performance in children with cerebral palsy treated with exoskeleton robotic training: a retrospective explorative analysis. Restor Neurol Neurosci. (2019) 37:239–44. 10.3233/RNN-180897 31177250

[23] Peri E Turconi AC Biffi E. Effects of dose and duration of Robot-Assisted Gait Training on walking ability of children affected by cerebral palsy. Technol Heal Care. (2017) 25:671–81. 10.3233/THC-160668 28436398

[24] Pierce SR Lauer RT Shewokis PA Rubertone JA Orlin MN. Test-retest reliability of isokinetic dynamometry for the assessment of spasticity of the knee flexors and knee extensors in children with cerebral palsy. Arch Phys Med Rehabil. (2006) 87:697–702. 10.1016/j.apmr.2006.01.020 16635633

[25] Ammann-Reiffer C Bastiaenen CHG Meyer-Heim AD Van Hedel HJA. Lessons learned from conducting a pragmatic, randomized, crossover trial on robot-assisted gait training in children with cerebral palsy (PeLoGAIT). J Pediatr Rehabil Med. (2020) 13:137–48. 10.3233/PRM-190614 32444573 PMC7458505

